# Taxonomic, Phylogenetic and Functional Diversity Behave Differently Under Disturbance Pressure and Complex Land‐Use History: Assembly Rules in Grassland Communities

**DOI:** 10.1002/ece3.73075

**Published:** 2026-02-12

**Authors:** Lucia Doni, Ian Briozzo, Bruno E. L. Cerabolini, Michele Dalle Fratte, Maria Guerrina, Luigi Minuto, Mauro G. Mariotti, Gabriele Casazza

**Affiliations:** ^1^ Department of Earth, Environmental and Life Sciences (DISTAV) University of Genoa Genova Italy; ^2^ Department of Biotechnologies and Life Sciences University of Insubria Varese Italy

## Abstract

Grazing and land abandonment trigger ecological succession and can affect plant communities by determining the relative importance of ecological assembly rules. A thorough understanding of these processes requires the implementation of taxonomic, phylogenetic, and functional diversity, along with knowledge of how they relate to each other in response to disturbance. We carried out survey on 120 plots and calculated taxonomic, phylogenetic, and functional diversity, the diversity's dimensionality, as well as the community weighted means to detect species functional response to changes in land‐use. Extensive grazing supported highest taxonomic and phylogenetic diversity. Whereas intense grazing had lowest diversity values. Abandoned grasslands resulted in differences between time periods, with past abandonment decreasing in diversity as succession advances. Functional diversity weakly varied among land‐use categories, yet the CWM analysis highlighted an increase in conservative resource‐use strategies through succession, and avoidance mechanisms with an increase in acquisitive traits in grazed communities. The importance of metrics in explaining the variation of the biodiversity space varied according to land‐use categories, where to the diversity of intensive grazing and past abandonment contributed most phylogenetic diversity, for intermediate grazing and recent abandonment the variation was explained mostly by functional diversity, and we found equal contribution between phylogenetic and functional diversity in extensive grazing. Our study suggests that reality is more complex than the simple paradigm that mechanisms of habitat filtering and limiting similarity lead to less and more diverse communities, highlighting the importance to treat the three diversity components as complementary. This knowledge supports management practices in grasslands experiencing grazing intensification or abandonment, especially in protected areas where legislation imposes responsibility for conservation action.

## Graphical abstract

Grasslands in the Alps are undergoing land‐use change triggering ecological succession and affecting community composition. Taxonomic, phylogenetic, and functional diversity are used as complementary information to infer on community assembly processes, supporting conservation practices.

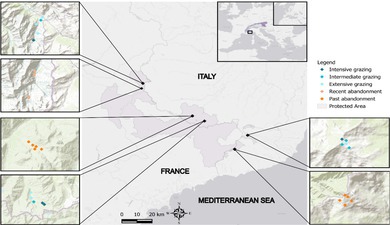

## 1 Introduction

Semi‐natural grasslands are highly dynamic systems determined by long‐term agro‐zootechnical activities (Bullock et al. 2011). However, during the last decades, many areas of Europe experienced the abandonment of traditional pastoral activities (Bohner et al. 2019), especially in more remote areas, which led to a drastic reduction of their surface and to changes in their floristic composition (MacDonald et al. 2000). At the same time, the most easily accessible pastures are increasingly heavily loaded with livestock and are, therefore, widely exposed to the risk of degradation and eutrophication (Tasser and Tappeiner 2002). Thus, semi‐natural grasslands are suitable to study diversity patterns of both currently and historically grazed plant communities leading to an improved comprehension of the consequences of land‐use change in alpine ecosystems (Kuhn et al. 2020) especially in protected areas where legislation imposes responsibility for conservation actions.

The spatial and temporal variation in grazing on high‐altitude pastures can differently drive plant communities' assembly, influencing landscape complexity and heterogeneity (Van der Maarel 2005). The disturbance and stress intensity can together affect species aggregation and coexistence in the community (Beyns et al. 2025; Cadotte and Tucker 2017; Maire et al. 2012; Munoz et al. 2014) which are recognized to be largely driven by the simultaneous action of two main processes. On the one hand, species coexistence is enhanced by species phenotype differences when competing for limited available resources (i.e., *limiting similarity hypothesis*; Macarthur and Levins 1967). On the other hand, environmental pressures may favor species capable of coping with disturbance or stress, leading to species sorting according to their tolerance capabilities (*habitat filtering hypothesis*; Kraft et al. 2015). The interplay between ecological niche availability and disturbance pressure can determine the relative importance of these two processes in plant communities (Cadotte and Tucker 2017; Maire et al. 2012). Along a disturbance gradient of grazing pressures, it is well established that biodiversity is higher in extensively managed grasslands (i.e., low‐input maintenance with low to no fertilizer application, lower grazing intensity, and infrequent mowing; Tiainen et al. 2020) due to reduced disturbance and an increase in the availability of ecological niches (Isselstein et al. 2005; Plantureux et al. 2005; Tiainen et al. 2020; Van Den Pol‐Van et al. 2019). Moreover, ecological succession and land‐use history also determine the relative importance of the assembly processes influencing communities' diversity (Connell and Slatyer 1977). During the early stages of vegetation succession after disturbance release, the effects of disturbance might persist, thus still acting as a filter in the process of species sorting (Purschke et al. 2013). Meanwhile, dispersal‐related processes drive successive changes in community composition and structure (Baasch et al. 2009). As succession advances, the role of habitat filtering might weaken over time, leaving space to competitive interactions to rule out species aggregation and coexistence while dispersal limitation rates decrease (Belyea and Lancaster 1999; Chang and HilleRisLambers 2016). The understanding of the above‐mentioned processes unravels the complexity of plant diversity in communities, which is crucial to predict vegetation dynamics in light of continuous land‐use changes (i.e., urbanization, afforestation, abandonment, agricultural intensification, etc.; Huyghe et al. 2014; Pazúr et al. 2024) in grassland habitats.

Quantification of taxonomic diversity is a groundbreaking method in ecology used to understand and know ecosystems' composition and structure. Species occurrence and abundance within a community provide information on the spatial and temporal heterogeneity of habitats (Swenson et al. 2012). This knowledge can be used to implement complementary methods for a deeper evaluation of other components of diversity, such as the evolutionary history, that is, phylogenetic diversity, and the functional role of species, that is, functional diversity (Srivastava et al. 2012). Patterns of phylogenetic and functional diversity within communities vary according to the disturbance intensity and land‐use change (Purschke et al. 2013; Tuo et al. 2024), and, therefore, they can allow us to ascribe the relative importance of assembly processes under specific habitat conditions (Grime 2006; Schroeder et al. 2024; Weiher et al. 1998). Hereafter, the use of metrics such as phylogenetic and functional distances can be used to assess whether community assembly results in non‐random assemblages and, therefore, driven by deterministic ecological mechanisms (Cadotte and Tucker 2017). In general, phylogenetic/functional convergence is expected when habitat filtering is the dominant process (Baraloto et al. 2012; Cavender‐Bares et al. 2004), whereas phylogenetic/functional divergence suggests the dominance of mechanisms that limit similarities (Ricotta and Moretti 2011). Moreover, taxonomic and phylogenetic diversity have been used explicitly or implicitly as a proxy for trait diversity based on the concept that more species being distantly related in a community should accumulate greater variation in ecological traits influencing species co‐existence (Fleishman et al. 2006; Le Bagousse‐Pinguet et al. 2019; Tucker et al. 2018; Webb et al. 2006). However, many studies have extensively reported that such correlation is dependent on many case‐specific factors, such as the habitat, landscape history, and environmental variability considered, thus implying a decoupling between these diversity components (see Cadotte et al. 2019; Carvalho et al. 2020; E‐Vojtkó et al. 2023; Hähn et al. 2025; Pavoine et al. 2013). Consequently, it is important to consider all facets of diversity and their relationship to fully grasp the reverberations of land‐use change on the diversity of ecological communities (Devictor et al. 2010).

To the best of our knowledge, only a few recent studies aimed at quantifying changes in all components of diversity along a gradient of grazing intensity and after abandonment of grazing activities (but see Sheng et al. 2023). In this study, we aim to better understand the main processes that drive community composition by using vegetation data collected from different grazing regimes and periods after grazing abandonment in the Southern‐Western Alps, and specifically to investigate: (i) the pattern of plant diversity components related to the gradient of grazing disturbance and the two periods of abandonment; (ii) the principal variation in functional traits in plant communities as a response to land‐use change; and (iii) the covariation among diversity components and how the relationship among them changes under different land‐use categories.

## 2 Materials and Methods

### 2.1 Study Area and Sampling Design

**FIGURE 1:**
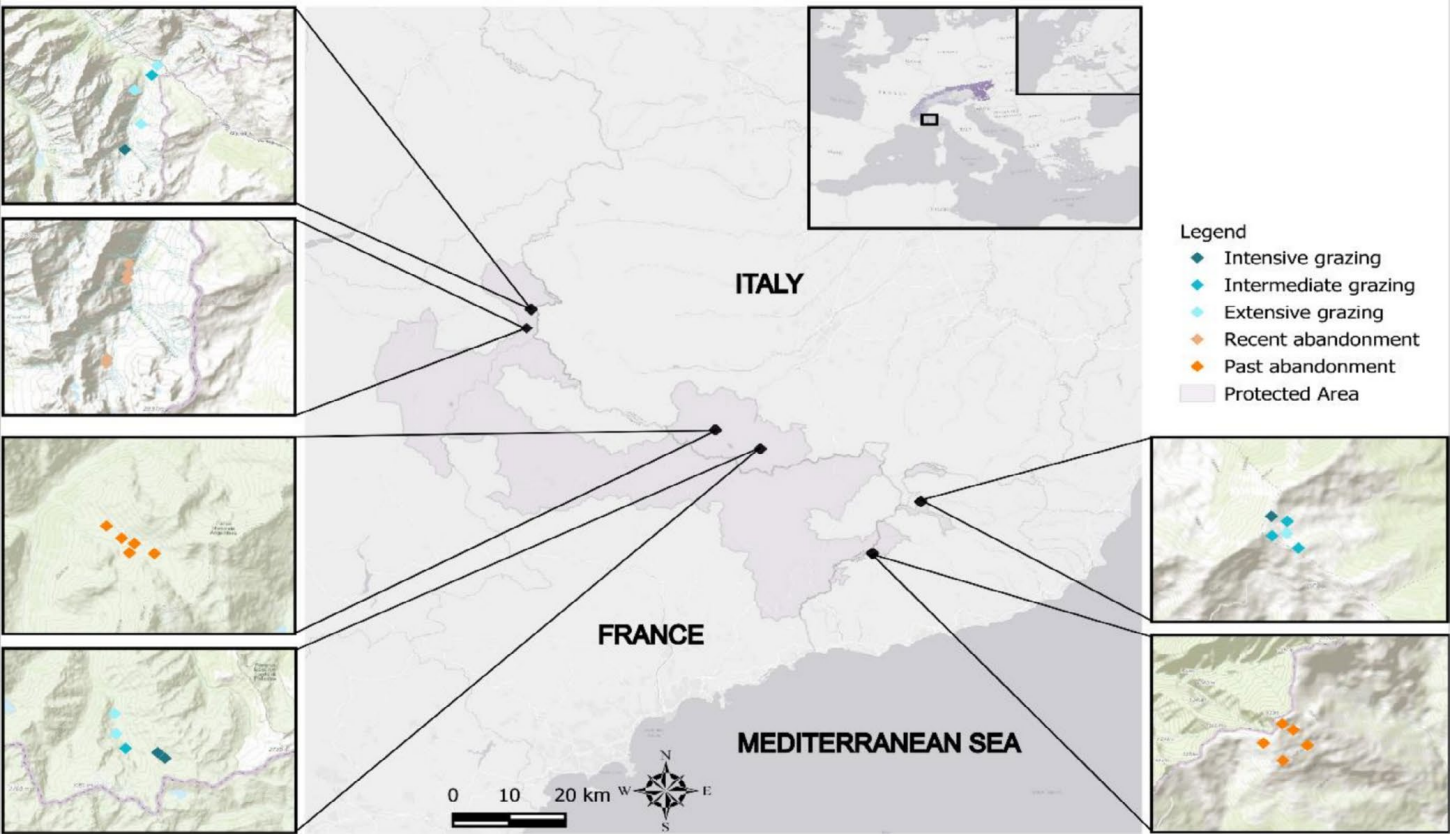
Sampling sites in the three protected areas (in light purple) comprising the Parco Naturale Regionale Alpi Liguri, Parco Naturale Alpi Marittime, Parc national du Mercantour. Diamond symbols indicate grazing sites under different grazing pressures and land abandonment: Extensive (light green‐cyan), intermediate (medium green‐cyan), and intensive (dark green‐cyan), recent abandonment (light orange) and past abandonment (dark orange).

The study was carried out in semi‐natural grasslands characterized by historical pastoral practices and land abandonment located between 1500 and 2200 m. a.s.l. in three protected areas of the SW Alps (Figure 1). The seminatural grasslands included in this study are mainly composed of habitats included in the European Habitats Directive 92/43/EEC such as acidophilic thermophilic subalpine grasslands (6150) and calciphile alpine and subalpine grasslands (6170), as well as priority habitats (EC 2013) like semi‐natural dry grasslands and scrubland facies on calcareous substrates (Festuco‐Brometalia; 6210*), and species‐rich *Nardus* grasslands, on silicious substrates in mountain areas (6230*). We identified grazed grasslands based on three degrees of livestock (sheep and cows) grazing pressure (i.e., extensive, intermediate and intensive) and abandoned grasslands based on two periods of abandonment: (i) less than 10 years, denominated as recent abandonment; and (ii) more than 10 years, denominated as past abandonment. We attributed the degree of grazing pressure to each grazed site according to the management plans of each protected area based on the calculation of the *optimal cattle load* defined as follows:

$$\mathrm{OL}=\frac{\left(\mathrm{SP}\right)\left(A\right)\left(\mathrm{kUt}/100\right)\;}{\left(I_{d}\right)\left(T\right)}$$

where kUt is a coefficient of utilization of the pastureland (% of the relationship between ingested phytomass and the phytomass present on the pasture before the pasture season begin), SP is the standardized production of grass of a given pastureland in a given year (kg ss/ha), *I*_d_ is the daily ingestion of grass by cattle, *A* is the unit area, and *T* the time period (express in days) of the grazing season.

We collected information on abandoned sites and the time since grazing cessation thanks to the ranger's knowledge in charge of high‐altitude pastures and their resource use. As found by Albert et al. (2014), what they defined as young succession (< 10 years) and middle‐aged succession (10–20 years) are suitable to detect changes in species composition and functional groups throughout spontaneous succession. Therefore, we retained the abandoned sites selected for this study as representative of earlier and later phases of ecological succession.

### 2.2 Data Collection

We carried out 120 vegetation relevés (4 m^2^ plots) using a stratified random sampling approach (Angelini et al. 2016). Vegetation data were gathered from May to August 2022. All samplings were performed before grazing to allow the detection of species in optimal conditions. For the analysis, we used taxa identified at the species level (*n* = 292). The nomenclature followed Pignatti (2017–2019). Phylogenetic data were obtained from the updated megaphylogeny of Angiosperms from Zanne et al. (2014) published by Qian and Jin (2016). Missing species were randomly added to their corresponding genera by using the *add.random* function in the *phytools* R package (Revell 2024). To account for uncertainties in the phylogenetic placement, the randomization procedure was repeated iteratively to obtain *n* = 1000 alternative topologies, which were used to compute averaged values for phylogenetic diversity metrics. For functional analysis we selected six plant traits representative of resource allocation strategies, capability of dispersal and regeneration rates, and the competitiveness of plant species (Díaz et al. 2016), as follows: specific leaf area (SLA), leaf dry matter content (LDMC) and leaf nitrogen content (LNC) for the economic spectra, as well as leaf area (LA), plant height (PH) and seed mass (SM) for the plant/organ size spectra. Trait data were mostly collected from the FIFTH (Cerabolini et al. 2010) and LIFTH (Dalle Fratte et al. 2021) databases (*n* = 1075), integrated with data (*n* = 462) from the available online database TRY (Kattge et al. 2020). The FITH and LIFTH databases can be used as proxies in this study as they are collections of plant traits of mostly alpine plant species. To complete missing data for LDMC (*n* = 35), SLA (*n* = 49), LA (*n* = 61), LNC (*n* = 39), and SM (*n* = 31), we applied taxonomic (family and genus) based gap filling, using the function *Gap‐Filling* of the *BHPMF* R package (Fazayeli et al. 2015). Gap filling was applied using all data from the Authors' datasets (FIFTH + LIFTH, *n* = 1746) to include a greater number of taxa, allowing greater prediction accuracy. We finally obtained a matrix of 1752 trait values. We relied on the abovementioned databases to collect data on functional traits since local measurements of traits are very time‐consuming (Ren et al. 2023). We are aware that intraspecific trait variability (ITV) affects functional diversity, but we applied the central assumption of “comparative ecology” which implies that trait variability within species is smaller than the differences between species (Westoby et al. 2002). The dataset used for taxonomic, phylogenetic, and functional analysis is available at https://doi.org/10.5281/zenodo.15746326.

### 2.3 Diversity Metrics

We measured taxonomic diversity for each plot with the Simpson index (*D*; Simpson 1949) by using the *spe* function implemented in the *vegan* R package (Oksanen et al. 2022). For phylogenetic (raoD) and functional diversity (raoQ), we used the Rao's Quadratic Entropy (*Q*: Rao 1982) calculated with the *raoD* function in the *picante* R package (Kembel et al. 2020), and with the *dbFD* function in the *FD* R package (Laliberté et al. 2014), respectively. They are known to perform well for detecting both lineage and trait convergence/divergence (De Bello et al. 2009; Ricotta and Moretti 2011). We estimated the community weighted mean (CWM; Garnier et al. 2004) based on abundance‐weighted data of all six selected traits with the *dbFD* function in the *FD* R package. The CWM can inform on the relationships between species traits and land‐use change.

### 2.4 Phylogenetic Signal

We used the phylogeny to test the phylogenetic signal (PS) of each selected trait to investigate whether closely related species tend to resemble one another. We calculated Blomberg's K (*K*; Blomberg et al. 2003) which assumes the classic Brownian Motion (BM) evolutionary model using the function *phyloSignal* implemented in the *phylosignal* R package (Keck 2025). Values can range between 0 and > 1, where values close to 0 indicate no phylogenetic signal (i.e., close relatives are not more similar than distant relatives), whereas values close to 1 indicate trait evolution according to BM. All six functional traits showed neither nor low PS (SLA‐*K* = 0.004; LDMC‐*K* = 0.005; LNC‐*K* = 0.003; LA‐*K* = 0.08; SM‐*K* = 0.02; PH‐*K* = 0.09) where only LA resulted statistically significant (*p* < 0.05).

### 2.5 Standard Effect Size

We were interested in detecting signals of habitat filtering and limiting similarity as potential mechanisms influencing community assembly along a gradient of grazing pressure and along ecological succession triggered by abandonment of pastures. Therefore, we calculated the standard effect size (SES), which is independent from species richness (Pavoine and Bonsall 2011) for both phylogenetic (raoD_SES_) and functional (raoQ_SES_) diversity as (observed diversity − mean expected diversity)/SD. We used the null model approach, creating 999 randomizations by shuffling the tips across the phylogenetic tree, and 999 randomizations of trait data, for phylogenetic and functional diversity, respectively. The SES values indicate to what extent communities are phylogenetically/functionally clustered (negative values) or dispersed (positive values) than expected by random. Moreover, we applied the trait‐based null model approach (999 permutations) to calculate the SES of each CWM to assess the strength of the effect of land‐use change on plant communities. The standard effect size of the CWMs (CWM_SES_) quantifies how much the observed CWM differs from what would be expected by chance.

### 2.6 Diversity Dimensionality

We wanted to determine the relationship among diversity's components in relation to land‐use change. For this, we evaluated the dimensionality (Stevens and Tello 2014) of grassland plant communities for each land‐use category by means of diversity metrics (i.e., *D*, raoD, raoQ) to understand whether the structure of communities presents different patterns of dimensionality according to land‐use. Diversity dimensionality is based on the amount of information needed to effectively characterize the variation presented in a community represented by the degree of complementarity among diversity's components. We, then, expanded the operationalization by integrating the importance value (IV) proposed by Nakamura et al. (2020) described as the amount of variation, or importance, that each diversity metric presents in fundamental biodiversity space. Therefore, we first calculated the evenness of eigenvalues (EE), based on the outputs of the PCA with a correlation matrix of diversity metrics, and estimated using Camargo's evenness index (Camargo 1993). Dimensionality values which approach 1 (high EE value) indicate high complementarity among diversity metrics, that is several dimensions are important to describe variation in community diversity. Conversely, dimensionality values approaching 0 (low value of EE) indicate high redundancy and correlation among metrics, that is a few dimensions of diversity are needed to describe diversity patterns. Then, we calculated the IV for each metric of diversity of each land‐use category obtained through the sum of values resulting from the multiplication between the variation of each principal component and the correlation of the metrics with that component. Low and similar IVs indicate high redundancy in metric importance, whereas high IVs denote low redundancy between metrics; that is, these metrics best describe the diversity variation in the community. To quantify the redundancy in the fundamental diversity space, we calculated an evenness metric (using Camargo's equitability index) using IVs for all metrics. High IVs evenness values indicate the contribution of more metrics to EE variation (Nakamura et al. 2020).

### 2.7 Statistical Analysis

Since functional traits have differing unit scales, we log‐transformed the data to reduce skewness of measurement variables. Furthermore, we tested for overall differences for *D*, raoD, raoQ, and CWMs by applying a Kruskal–Wallis test, using the non‐parametric Wilcoxon post hoc test for pairwise comparisons. For raoD_SES_, raoQ_SES_, and CWM_SES_, one‐sample *t*‐test was calculated to determine whether mean values were significantly different from zero.

## 3 Results

### 3.1 Diversity Analysis

**FIGURE 2:**
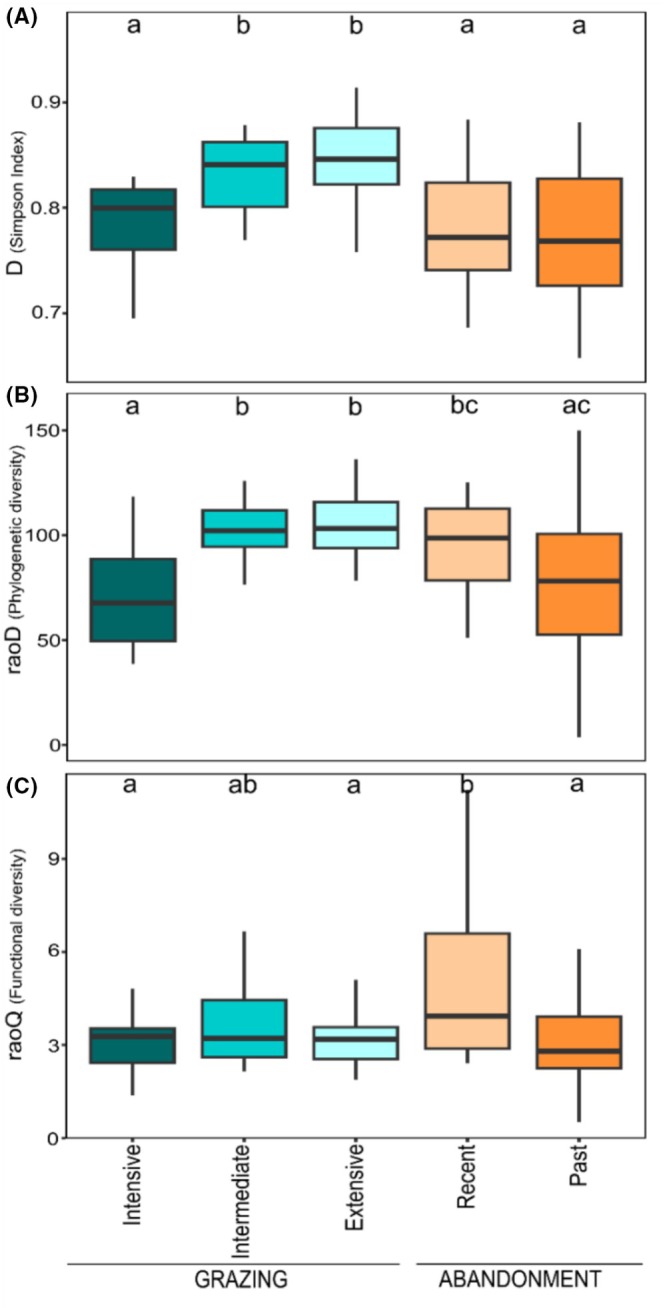
Diversity analysis of taxonomic (*D*; Simpson index), phylogenetic (raoD; Rao quadratic entropy), and functional (raoQ; Rao quadratic entropy) diversity for each land‐use scenario. Starting from the top: Taxonomic diversity (A), phylogenetic diversity (B), and functional diversity (C). Different letters indicate statistically significant differences (*p* > 0.05).

**FIGURE 3:**
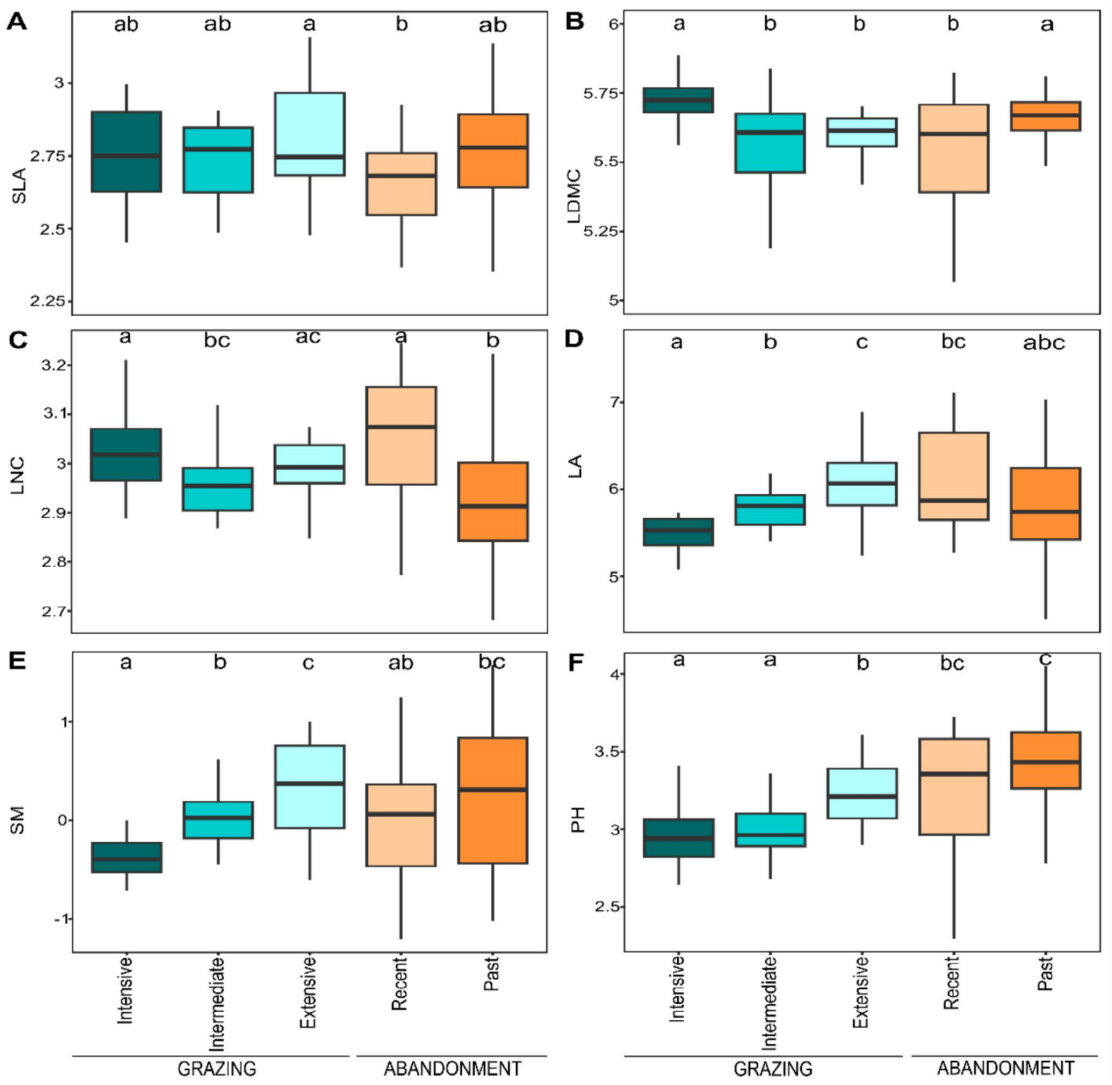
The Community Weighted Means (CWMs) of the economic spectra: Specific leaf area (SLA), leaf dry matter content (LDMC), leaf nitrogen content, and of the plant organ/size spectra: Leaf area (LA), seed mass (SM), plant height (PH), for each land‐use scenario. Starting from the top left: SLA (A), LDMC (B), LNC (C), LA (D), SM (E), and PH (F).

For taxonomic diversity (*D*; Figure 2A), we generally detected significantly higher values in grazed than in abandoned sites. Extensive grazing had the highest values (mean [*M*] = 0.84, standard error [SE] = 0.06), followed by intermediate grazing (*M* = 0.83, SE = 0.04). In contrast, intensive grazing resulted in similar values to recent and past abandoned sites (*M* = 0.79, SE = 0.045; *M* = 0.78, SD = 0.065; *M* = 0.73, SD = 0.148, respectively). Phylogenetic diversity (raoD; Figure 3B) was higher in intermediately and extensively grazed sites (*M* = 101 for both, SE = 1.68 and 2.22, respectively), but not statistically different from recent abandonment (*M* = 94.5, SE = 2.53). Intensive grazing and past abandoned sites had the lowest phylogenetic diversity values (*M* = 72.4, SE = 3.04; *M* = 79, SE = 6.26, respectively). For functional diversity (raoQ; Figure 3C), we detected higher values in recently abandoned sites (*M* = 5.1, SE = 1.33), followed by intermediately grazed ones (*M* = 3.73, SE = 0.87), which, however, did not show significant differences compared to the other land‐use categories. Intensive grazing, extensive grazing, and past abandonment had the least values of functional diversity (*M* = 3.11, SE = 0.55; *M* = 3.12, SE = 0.45; and *M* = 3.08, SE = 0.87, respectively).

### 3.2 CWMs

In the community weighted mean (CWM) analysis, traits related to the economics spectra (i.e., leaf dry matter content, LDMC; specific leaf area, SLA; leaf nitrogen content, LNC) resulted relatively similar among land use scenarios, with few weak tendencies detected in the ecological strategies of species (Figure 3A–C). Regarding SLA, only extensively grazed sites resulted significantly higher than recent abandonment (*M* = 2.80 and 2.66, SE = 0.11 and 0.1, respectively) while the rest of land‐use categories were similar among each other. LDMC had the highest values in intensively grazed and past abandoned sites (*M* = 5.71 and 5.67, SE = 0.04 and 0.05, respectively; Figure 3B), opposed to all the other land‐use categories. Lastly, we detected the lowest LNC values in past abandoned sites (mean = 2.92, SE = 0.7) although not significantly different from intermediate grazing (mean = 2.96, SE = 0.04; Figure 3C). The highest values of LNC were observed at intensive grazing and recently abandoned sites (mean = 3.02 and 3.04, SE = 0.05 and 0.08, respectively). The traits related to the plant/organ size spectra (i.e., leaf area, LA; plant height, PH; seed mass, SM) were found to be more significantly different across land‐use categories compared to the economics spectra. In particular, the values of plant/organ size significantly decreased with increasing grazing intensity, while they did not exhibit significant differences between different abandonment scenarios (Figure 3D–F). In abandoned sites, LA and SM were not significantly different from grazed ones, while we recorded an increase of PH in abandoned sites (*M* = 3.25, SE = 0.23 for recent abandonment; *M* = 3.44, SE = 0.16 for past abandonment) with respect to intensively and intermediately grazed ones (*M* = 2.93 and 2.97, SE = 0.13 and 0.13, respectively).

### 3.3 Phylogenetic, Functional and CWMs Standard Effect Size

**FIGURE 4:**
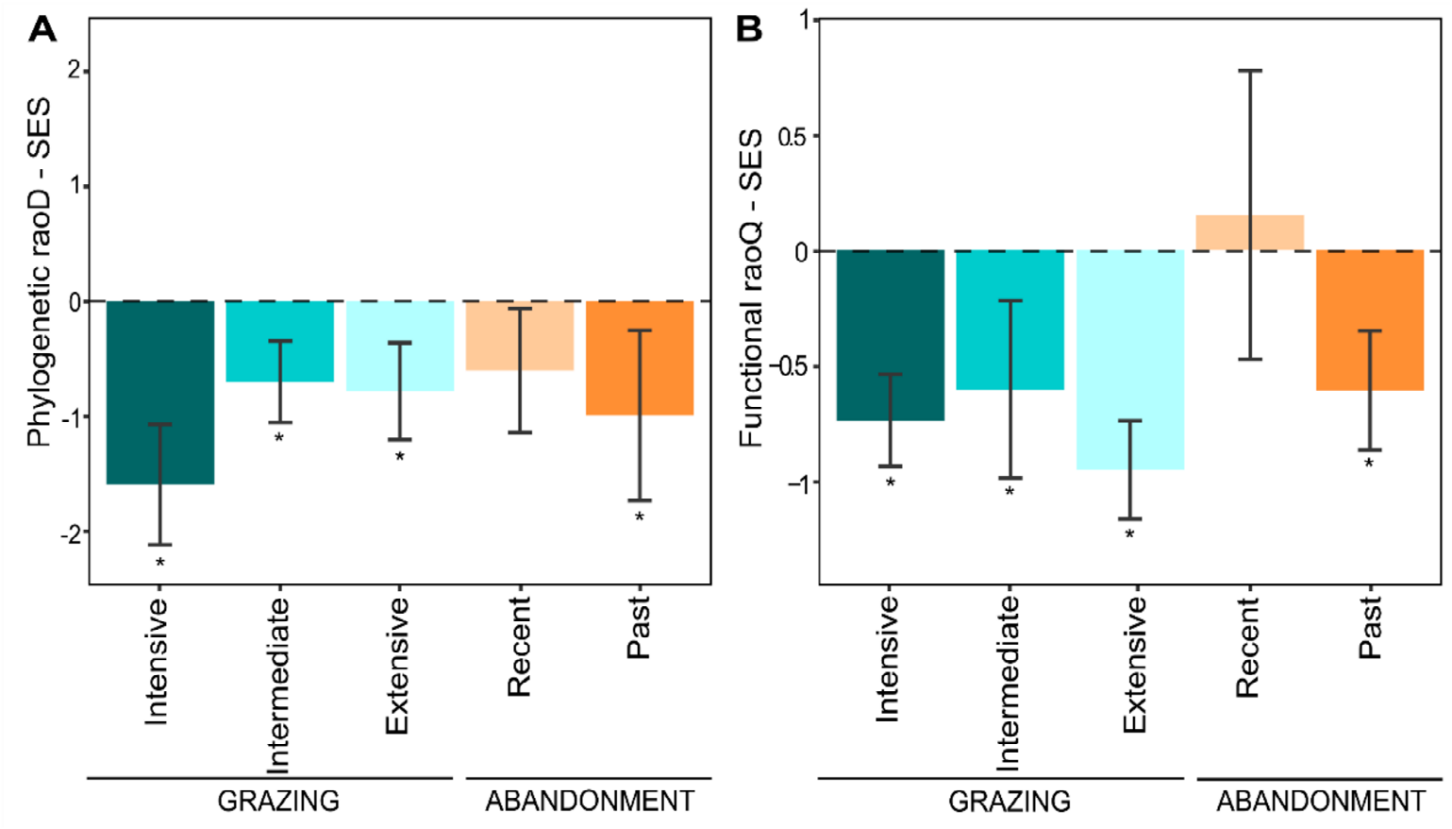
Phylogenetic (A) and functional (B) standard effect size (SES). The asterisk (*) indicates significant values different from 0 (*p* < 0.05).

Mean values for standard effect size (SES) ranged between −0.79 and −1.77 for raoD_SES_ (Figure 4A), and between 0.17 and −1.06 for raoQ_SES_ (Figure 4B). For most of the land‐use categories, phylogenetic and functional diversity showed similar trends of lineages and trait convergence (negative values; *p* > 0.05). Differently, recent abandonment resulted in a decoupled trend of raoD_SES_ and raoQ_SES_ with a tendency of positive values of functional (trait overdispersion; mean = 0.17) but negative values of phylogenetic (lineages convergence; mean = −0.67) SES. For both raoD_SES_ and raoQ_SES_, values did not significantly differ from zero for recent abandonment, indicating random patterns of species aggregation.

**FIGURE 5:**
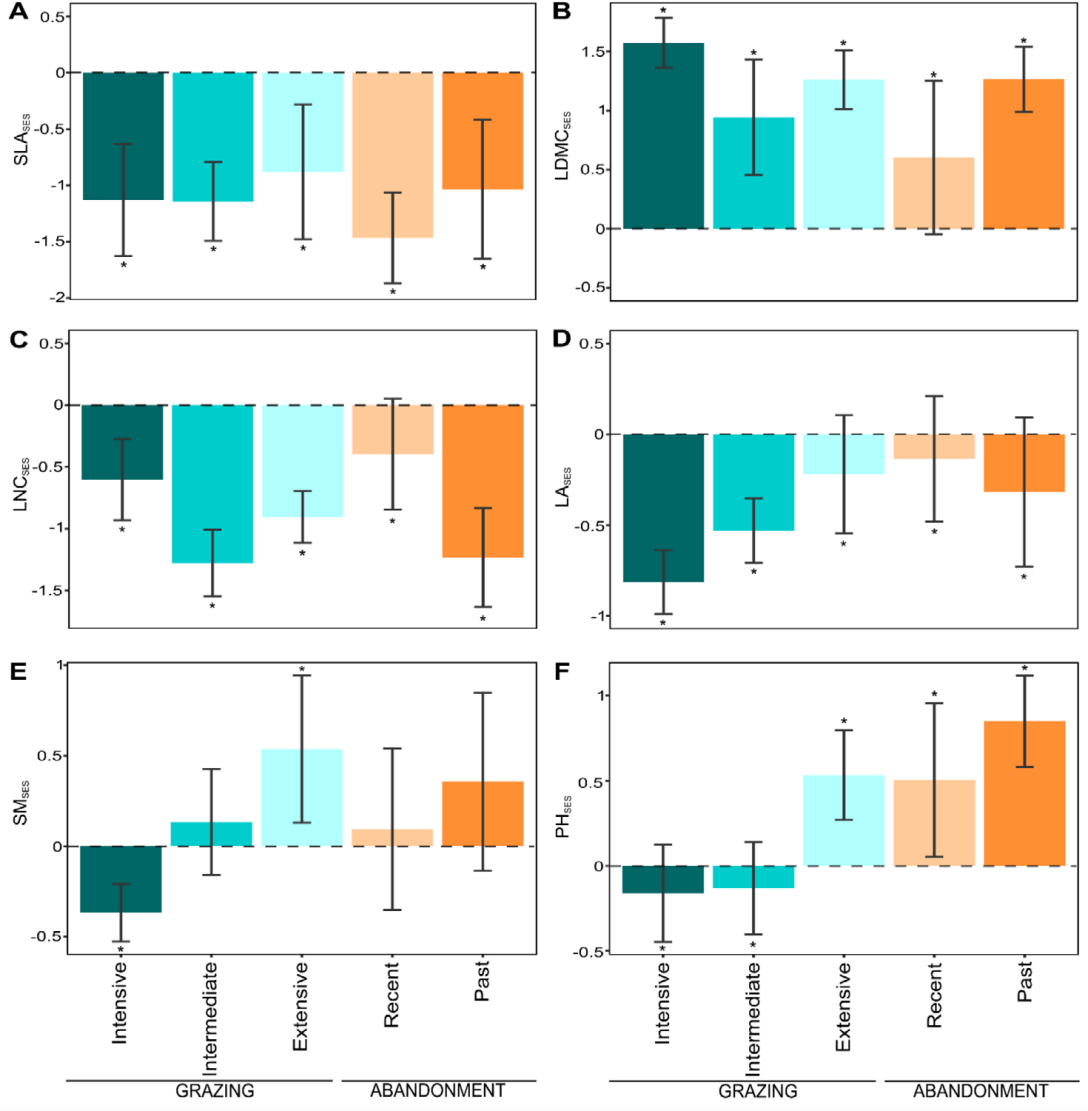
Standard effect size (SES) of CWMs of each functional trait. Starting from the top left: Specific leaf area (A), leaf dry matter content (B); leaf nitrogen content (C); leaf area (D); seed mass (E); plant height (F). The asterisk (*) indicates significant values different from 0 (*p* < 0.05).

The CWM_SES_ (Figure 5) showed values of traits related to the economic spectrum to be lower than expected by chance for all land‐use categories in the case of SLA_SES_ (range from −0.88 to −1.47, *p* < 0.05; Figure 5A) and LNC_SES_ (range from −0.4 to −1.28, *p* < 0.05; Figure 5C), and higher in the case for LDMC_SES_ (range from 0.6 to 1.57, *p* < 0.05; Figure 5B). Also, in the plant organ/size spectra LA_SES_ (Figure 5D) resulted in values lower than expected by chance for all land‐use categories (range from −0.13 to −0.81, *p* < 0.05); whereas for SM_SES_ (Figure 5E) intensive grazing resulted in values lower than expected (mean = −0.37, *p* < 0.05), while extensive grazing was observed with the highest values (mean = 0.53, *p* < 0.05) of SES compared to all other categories, which resulted in an average positive value (range from 0.09 to 0.53) not significantly different from zero. Lastly, we detected positive PH_SES_ (Figure 5F) for extensive grazing (mean = 0.53; *p* < 0.05), recent abandonment (mean = 0.5; *p* < 0.05), and past abandonment (mean = 0.85; *p* < 0.05); meanwhile, intensive and intermediate grazing resulted in negative SES values (mean = −0.16 and mean = −0.13, respectively; *p* < 0.05).

### 3.4 Dimensionality of Biodiversity

In general, dimensionality values (EEs; Table 1) resulted in similar values among land‐use categories ranging between 0.57 and 0.79 indicating a medium to low correlation among metrics of diversity, thus medium to high complementarity among diversity's components. The first principal component of the PCA explained between 70.5% and 82.8% of the variation, principally represented by raoQ, whereas the second principal component accounted for between 17.25% and 29.5% with higher loadings for raoD. The load of *D* is more evenly distributed between the first and the second axes of the PCA. These trends were observed to be similar among land‐use categories despite past abandonment, which on the first axis *D* had a higher loading, whilst the second axis was mainly represented by raoD and raoQ.

**TABLE 1:** Evenness of eigenvalues (EE) and importance value (IV) for each land‐use category.

Land‐use	EE		IVs	
	*D*		raoD	raoQ
Intensive grazing	0.64	0.01	0.63	0.35
Intermediate grazing	0.72	0.004	0.12	0.87
Extensive grazing	0.65	0.04	0.48	0.48
Recent abandonment	0.79	0.01	0.24	0.74
Past abandonment	0.57	0.105	0.52	0.38

The IV analysis (Table 1) highlighted phylogenetic diversity (raoD) with the highest contribution to the variation in the fundamental space of diversity for intensive grazing and past abandonment (IV = 0.63; IV = 0.52, respectively). On the contrary, for intermediate grazing and recent abandonment, functional diversity (raoQ) best contributed to the variation (IV = 0.87; IV = 0.74, respectively). Lastly, extensive grazing resulted in equal importance values regarding raoD and raoQ (IV = 0.48). Taxonomic diversity (*D*) represented minimal variation in the fundamental space of diversity for all land‐use categories.

**FIGURE 6:**
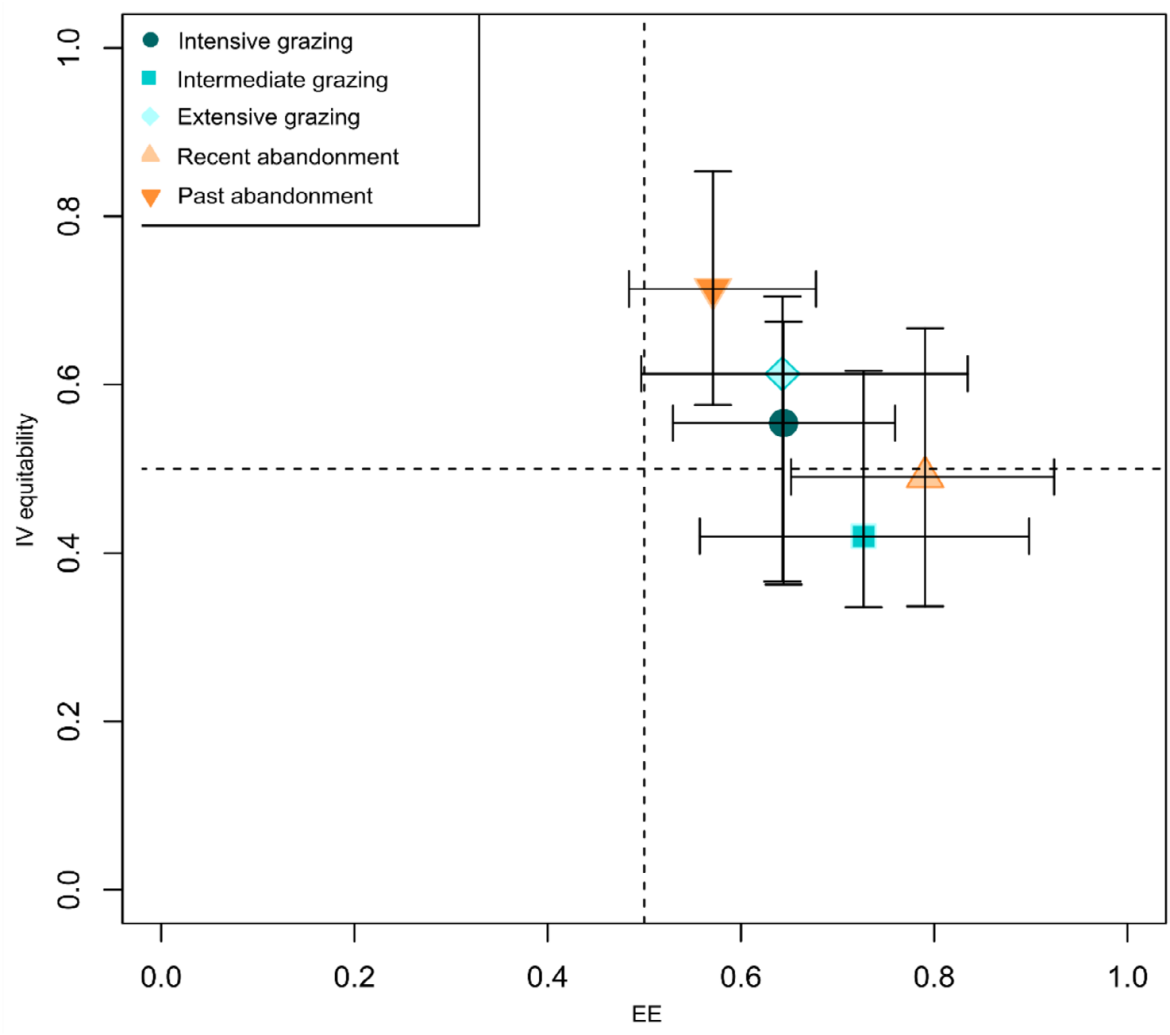
IVs evenness for each land‐use category. Error bars denote 95% confidence interval.

The integrated dimensionality measure (EEs + IVs; Figure 6) resulted in a general high EE for each land‐use category, especially for intermediate grazing and past abandonment, yet with higher variance for the IV equitability index. Although demonstrating a relatively high complementarity among metrics (high EEs), extensive grazing and past abandonment resulted in higher redundancy of metrics (high IV equitability), indicating more metrics that contribute to EE variation. The other land‐use categories were observed with lower values of IV equitability, thus detecting low redundancy among metrics.

## 4 Discussion

In this study, we investigated the effects of livestock grazing and land abandonment on semi‐natural grassland plant communities in the SW Alps. We used the three components of diversity to explore assembly rules in driving community composition of high‐altitude pastures, as well as to understand how the three components relate to each other under recurrent disturbance events and along ecological succession after grazing activities ceased. In general, we detected the highest values in taxonomic and phylogenetic diversity under low grazing pressure (i.e., extensive and intermediate categories). Conversely, functional diversity weakly varied with highest values recorded in recent abandonment. In extensively and intermediately grazed sites, the spatial heterogeneity in grazing, trampling, and dropping can create a mosaic of environmental patches over short distances, thus increasing the availability of microsites (Questad and Foster 2008). The latter allows the establishment of highly diverse species also favored by enhanced light accessibility or nutrient availability (Grime 2001). Nevertheless, the selective action of grazing filters species sharing similar functional traits. As grazing severity intensifies (i.e., intensive grazing), the number of environmental patches is reduced (i.e., increasing environmental homogeneity), and the intense pressure enables fewer species to survive and establish in the community. In fact, functional and phylogenetic convergence detected in grazed sites (Figure 4) indicate that grazing created a strong filter effect across the pressure gradient. The strong phylogenetic clustering for intensive grazing can be related to the loss of functionally and phylogenetically related species. Extensive and intermediate grazing had a weaker phylogenetic clustering suggesting a moderate exertion of filtering processes on communities (Kasprzyk et al. 2025) where the weaker effect of habitat filtering allows for more diverse lineages to enter the community causing higher phylogenetic diversity (Figure 2).

In recently abandoned sites, we detected a reduction of taxonomic diversity, a weak reduction of phylogenetic diversity, and an increase of functional diversity. Whereas in the past abandonment, plant communities showed a slight decrease in phylogenetic diversity and a strong decrease in functional diversity with respect to recent abandonment. Here, it should be addressed the differences in diversity between abandoned and grazed sites could also stem from their spatial distribution (Figure 1) of which distance range between ca. 1 and 3 km among the closest sites and between ca. 30 and 80 km among the farthest sites, thus possibly owing to slightly different regional species pools. Furthermore, the high variability recorded among plant communities at abandoned sites is likely due to the different extent and direction of change that can vary considerably with the history of grazing and abandonment, and the heterogeneous abiotic conditions characteristic of the semi‐natural grasslands of the SW Alps. During the early stages of ecological succession, because the biological legacy left by past grazing activities still act as a filter (Purschke et al. 2013), there is a gradual replacement of grazing‐tolerant species with first colonizers introducing new functional traits in the community. In fact, population turnover since abandonment can be slow as “remnants” of plant populations dominant during grazing may persist over time (Johansson et al. 2011; Tasser and Tappeiner 2002). During this stage, diversity and species abundance are mainly governed by processes related to chance and dispersal capability of species (Baasch et al. 2009; Zillio and Condit 2007), resulting in an excess of low‐abundant species in the community. This demographic stochasticity can reduce the similarity of competing species and increase niche differences among them (see Schwilk and Ackerly 2005; Tilman 2004). In fact, multiple strategies can be present in low abundance, thus increasing functional diversity in the community (Gyllenberg and Meszéna 2005). Furthermore, stochastic dynamics of community assembly are reflected by the absence of a clear pattern of divergence/convergence of traits and lineages among species in the community (Figure 4), suggesting a balance between limiting similarity and habitat filtering processes (Pavoine et al. 2010). Hence, our findings do not support habitat filtering as the major process driving community assembly during early succession but rather an equilibrium between the two. As succession advances, processes related to chance and dispersal capability of species weakened and competitive interactions become more important due to the increased match between the vegetation and the environment (Zaplata et al. 2013). The resulting establishment of fewer abundant, dominant species leads to the exclusion of the weaker competitors (Bolker et al. 2003), and phenotypic dissimilarities are reduced due to the increased share in species' ecological strategies (Pacala and Tilman 1994). In fact, we observed functional and phylogenetic convergence with respect to the early stages of succession (i.e., recent abandonment). The latter is in contrast to our expectation to find an increase in phylogenetic and trait overdispersion as succession advances (i.e., influence of limiting similarity mechanism). In fact, as argued by other studies, an increase in competitive interactions within a biological community does not always lead to trait and phylogenetic overdispersion (Cahill et al. 2008; Cavender‐Bares et al. 2004; Roscher et al. 2016). Yet, to affect phylogenetic structure traits involved in the assembly process need to be phylogenetically conserved (Bennett et al. 2013; Mayfield and Levine 2010), differently to our finding (i.e., no phylogenetic signal). Moreover, the trade‐off between colonizing ability and traits related to competition are core to succession processes (Tilman 2004) where species with high colonization ability arrive faster (i.e., early succession) but which persistence depend on the arrival of later‐colonizing species that generally own greater competitive ability (Roscher et al. 2016). Therefore, strong competitors dominate in later stages of succession, leading to functional and phylogenetic convergence (Gerhold et al. 2015). Overall, our results do not fully support the general expectation of an increase diversity at an intermediate level of disturbance (e.g., Moi et al. 2020) and a decrease along successional stage (e.g., Uchida and Ushimaru 2014) since the specific arrangement of species of a community, and the ecological mechanism involved thereby, is influenced by many factors and is not predictable via simple rules. Otherwise, they suggest that the three aspects of diversity are differently affected by grazing intensity and ecological succession. However, the degree of concordance between phylogenetic and functional diversity depends on the phylogenetic signal exhibited by the traits analyzed (Flynn et al. 2011; Swenson and Enquist 2009). Continuous traits, such as the ones used in this study, have a high phenotypic plasticity and so are easily affected by the environment, usually resulting in a low phylogenetic signal (Zhou et al. 2019). Also, combination of traits can weaken the relationship between phylogenetic and functional diversity in case of complex evolution models of traits (Murrell 2017; Tucker et al. 2018). In fact, not all traits are ecologically relevant, and some traits relate more directly to niche axes than others (Garnier et al. 2016). Therefore, increasing the number of traits could support the detection of more traits that are directly involved with response to grazing. Additionally, direct measurement of traits rather than relying on databases could increase the signal of the response to land‐use change, as local variation of species traits is accounted for.

Plant communities' functional traits differed mainly in the plant/organ size spectra, confirming that changes in grazing management have little change on CWM traits mostly related to plant size (Pakeman and Fielding 2020). In particular, the size spectra increased from intensive to extensive grazing. In the economic spectra, LDMC is significantly higher in intensively grazed and past abandoned plots. The remarked response of species in the plant/organ spectrum of traits was also confirmed by the CWM_SES_ analysis were mainly SM and PH resulted in tendencies higher and lower than expected depending on land‐use change. In general, plant species may respond to grazing by adopting one of these two mechanisms: avoidance or tolerance (Briske 1996). The avoidance consists of reducing plant size and moving toward a conservative resource‐use strategy as increase leaf dry matter content to reduce palatability (Jiang et al. 2023). The tolerance consists into adapting to an acquisitive resource‐use strategy as increasing specific leaf area and leaf nitrogen content to boost growth rate (Díaz et al. 2001). Our results suggest that in SW alpine grasslands plant species adopt an avoidance strategy in response to grazing pressure. The reduction in plant size (i.e., LA and PH) is probably caused by cattle feeding behavior that avoid small leaves as they require more feeding efforts (Díaz et al. 2001), in turn reducing competition for light and favoring the persistence of small species (Grime 2001; Westoby et al. 2002). The reduction in seed size was recorded in some previous works (e.g., Li et al. 2022) and could be a side effect of the reduction in plant size, since shorter species usually produce smaller seeds (Osem et al. 2006), resulting in a positive correlation between plant and seed size (Lavergne et al. 2003). Moreover, the increase of small‐seeded species (e.g., *Phleum rhaeticum* [Humphries] Rauschert, *Campanula scheuchzeri* Vill., *Anthoxanthum odoratum* L., *Cerastium arvense* L., *Plantago maritima* L., *Festuca rubra* L., *Nardus stricta* L.) with grazing pressure may be a strategy to cope with disturbance. In fact, the small‐seeded species produce a larger number of seeds (Smith and Fretwell 1974), heightening the seed persistence in the soil (Pakeman et al. 2002; Wu et al. 2015). As grazing pressure decreases and plant size increases resulting in higher competition for light, there is a shift from small‐seeded species to large‐seeded ones, as detected in extensive grazing and past abandonment. In shaded habitats, large seeds give a recruitment advantage because they usually produce larger and more vigorous seedlings than small ones (Silvertown 1981). The small decline in seed size observed in recent abandonment with respect to extensive grazing is likely related to community dynamics and recolonization by small‐seeded species. These have a large number of seeds that result in a larger number of colonization opportunities (i.e., higher seed rain; Leishman 2001). Moreover, the greater colonization potential of small seeds is also due to the reduced loss of seeds to predation and pathogens (Thompson et al. 1993).

The relatively high values of LDMC may be related to the low rate of evapotranspiration occurring in sub‐ and alpine ecosystems (Gardarin et al. 2014). Moreover, with decreasing temperatures conservative traits (e.g., LDMC) increase in alpine plant species (Henn et al. 2018). The lack of significant differences among extensive and intermediate grazing and recent abandonment scenarios might be explained by the relatively small alteration in herbivory pressure (at grazed sites) and the persistence of the biological legacy of past grazing activities during the early stages of succession (at abandoned sites). Nevertheless, the significantly higher values detected in intensive grazing are in line with avoidance strategy reducing both palatability and digestibility of plants and, in turn, the forage quality (Blumenthal et al. 2020; Pontes et al. 2007). The increase in LDMC detected in past abandonment is likely due to the increase in tall tussocks and graminoids abundance that usually have higher LDMC values (Akram et al. 2022).

Taken together, these results suggest that the main mechanism to cope with herbivory in the SW alpine grasslands is avoidance, mainly affecting plant size spectra. Our result contradicts a previous study detecting tolerance as the main mechanism in the primary high‐altitude grasslands in the Central Alps (Zanzottera et al. 2020). This disagreement may be related to dissimilarities in local climatic and environmental conditions between the two study areas, which are located in distinct parts of the Alps, resulting in different soil legacies concerning nutrient and water availability. In fact, the SW Alps have roughly half of the annual precipitation compared to the Central Alps (Isotta et al. 2014). The response of species to grazing strongly depends on moisture availability, where species select resource‐acquisitive and grazing‐tolerance strategies in wet and fertile habitats and resource‐conservative and grazing‐avoidance strategies in dry and less fertile ones (Zheng et al. 2015).

The dimensionality of grassland plant communities varied according to land‐use category. Overall, little correlation (EE; Table 1) was found among the three components of diversity meaning that to fully capture most of the information on grassland communities of the SW Alps we need to approach the quantification of diversity in a multidimensional manner, encompassing taxonomic, phylogenetic and functional elements (Liborio 2025; Lyashevska and Farnsworth 2012; Nakamura et al. 2020). The greatest disparity among land‐use categories was grasped by the IV metric. Phylogenetic diversity exhibited the highest amount of variation for intensive grazing and past abandonment, whereas functional diversity accounted for the largest variation in the case of intermediate grazing and recent abandonment. These observations might suggest that phylogenetic, in the first case, and functional, in the second one, capture more ecological variation within plant communities, respectively (Stevens and Gavilanez 2015). Extensive grazing is characterized by an increase in redundancy among metrics, especially between raoD and raoQ, indicating that the two metrics held a similar amount of information on biodiversity space (Nakamura et al. 2020). Interestingly, taxonomic diversity gave a minimum estimate of total diversity across land‐use categories suggesting the sole use of this metric is an incomplete surrogate for grassland plant communities (Wilsey et al. 2005; Paula‐Souza et al. 2023). We observed higher IVs evenness values (Figure 6) for past abandonment and extensive grazing indicating the contribution of more metrics to EE variation. Then, lower values of IV evenness observed in a descending order for intensive grazing, intermediate grazing and recent abandonment indicate that with an increase in complementarity among metrics fewer dimensions of diversity contribute to the biodiversity space. The general mismatch of dimensionality across land‐use categories is possibly related to the assembly of and coexistence among species in local communities (Stevens and Gavilanez 2015). In fact, we found a difference in the importance of dimension driving species sorting during the assembly process depending on land‐use change. For instance, tendencies of phylogenetic clustering and functional overdispersion in recently abandoned communities might rationalize incongruencies among dimensions of diversity, which can be enhanced when different processes structure biotic communities (Stevens and Gavilanez 2015).

In relation to our first research question, we concluded that assembly rules act in various ways on taxonomic, phylogenetic, and functional diversity, depending on disturbance pressures and stage of ecological succession. Our second research question investigated the patterns of CWMs in relation to land‐use change, and we observed that plant communities at grazed sites tend to acquire a conservative strategy in response to grazing, whereas as ecological succession advances, species move toward more competitive strategies. We also revealed the importance of the edaphic legacy to drive species strategies to land‐use change. Finally, regarding question three, the dimensionality analysis demonstrated a moderate to high complementarity among diversity dimensions across land‐use categories and variation among metric importance in explaining the biodiversity space. Overall, we can affirm the importance of treating the three diversity components as complementary information and not as proxies to infer the consequences of land‐use change on plant communities. Furthermore, our study supports the idea that reality is more complex than the simple paradigm that mechanisms of habitat filtering and limiting similarity lead to less and more diverse communities, respectively (Cadotte and Tucker 2017). Further studies could investigate the drivers of the incongruences among biodiversity indices in plant communities also in relation to dimensionality by considering multiple diversity scales (e.g., beta diversity). The boost in research advancement on the behavior of diversity components of biotic communities under different environmental and land‐use conditions can support decision‐making on the choice of conservation strategies and spatial prioritization planning.

## Author Contributions

**Lucia Doni:** conceptualization (equal), data curation (lead), formal analysis (lead), investigation (lead), methodology (equal), writing – original draft (lead), writing – review and editing (equal). **Ian Briozzo:** data curation (supporting), methodology (supporting). **Bruno E. L. Cerabolini:** methodology (supporting), writing – review and editing (equal). **Michele Dalle Fratte:** formal analysis (equal), methodology (equal), writing – review and editing (equal). **Maria Guerrina:** data curation (supporting), formal analysis (supporting), investigation (supporting), methodology (equal), writing – review and editing (equal). **Luigi Minuto:** investigation (supporting), methodology (supporting), writing – review and editing (equal). **Mauro G. Mariotti:** conceptualization (equal), funding acquisition (lead), project administration (lead), supervision (supporting), writing – review and editing (equal). **Gabriele Casazza:** conceptualization (equal), formal analysis (supporting), investigation (supporting), methodology (equal), supervision (equal), writing – original draft (supporting), writing – review and editing (equal).

## Funding

This work was supported by the European Union's Horizon 2020 research and innovation programme under the Marie Skłodowska‐Curie GA No. 101034449.

## Conflicts of Interest

The authors declare no conflicts of interest.

## Data Availability

Data are available in the Zenodo repository (https://doi.org/10.5281/zenodo.15746326).
